# A Voronoi-Diagram-Based Load Transfer Rule: An Application to Damage Evolution in Suddenly Loaded Arrays of Pillars

**DOI:** 10.3390/ma18235425

**Published:** 2025-12-02

**Authors:** Zbigniew Domański, Tomasz Derda

**Affiliations:** Department of Mathematics, Czestochowa University of Technology, Dąbrowskiego 69, 42-201 Częstochowa, Poland; zbigniew.domanski@pcz.pl

**Keywords:** array of pillars, failure evolution, fibre bundle model, load sharing rule, Voronoi tessellation

## Abstract

Arrays of pillars fabricated on flat substrates belong to a class of multicomponent systems composed of many interconnected elements functioning in parallel. Under sudden loading, their load-bearing capacity depends not only on the intrinsic strength of individual pillars but also on the mechanism by which loads released from crushed pillars are redistributed to surviving ones. Following the initial application of load, pillars with thresholds below the applied stress collapse, and their loads are transferred according to a prescribed load-sharing rule, triggering bursts of failures. These bursts may either drive the system to complete collapse or stabilise it in a partially damaged configuration. In this work, we introduce a novel phenomenological load transfer rule that explicitly incorporates the system geometry and the elastic properties of the substrate. When the pillars are placed on a homogeneous, isotropic substrate and crushing occurs instantaneously, the redistributed loads are transferred to intact pillars located within the Voronoi cells defined by the ones failed simultaneously. Since the locations of crushed pillars evolve during the loading process, the Voronoi load sharing (VLS) rule is inherently dynamic rather than static. Within the fibre bundle model framework, we simulate suddenly loaded pillar arrays to evaluate their overall strength and to characterise the spatio-temporal evolution of damage under the VLS rule. These findings are systematically compared with those obtained from other established load-transfer rules.

## 1. Introduction

A wide range of engineering systems are inherently multicomponent, consisting of large assemblies of functionally identical units operating in parallel. Under external loading, these units act collectively to perform their assigned function. However, when one unit becomes overloaded and fails, its load must be redistributed among the surviving units. This redistribution can, in turn, overload additional units, triggering further failures. Such cascading sequences progressively degrade system performance and may eventually evolve into avalanches of failures. In extreme cases, the avalanche becomes self-sustaining, leading to catastrophic collapse of the entire system.

In this study, we consider arrays of pillars as representative mechanical systems. Specifically, arrays of micro- and nano-pillars play an important role in a variety of practical and potential applications [1,2,3,4]. Materials and components at these scales typically demonstrate enhanced strength and toughness compared to macroscopic systems, while simultaneously displaying pronounced sample-to-sample fluctuations and non-trivial size effects [5,6,7,8,9]. Consequently, the overall strength and integrity of an array are governed by several key factors, including the spatial arrangement of pillars, their individual strength thresholds, the external loading protocol, and the adopted load-transfer rule. Among these, the choice of loading scheme and the specification of the load-transfer rule are of primary importance.

Our model is based on the Fibre Bundle Model framework, a classic statistical mechanics approach widely used to describe failure in multicomponent systems [10,11,12,13]. Within this framework, two loading schemes are typically employed: quasi-static and sudden loading. In the quasi-static scheme, the external load is increased incrementally, causing the weakest pillar to fail at each step. After each failure, the released load is redistributed among the remaining intact pillars, and the system relaxes into a new stable configuration. This process continues until the prescribed total load *F* is reached. In contrast, under sudden loading, the entire load *F* is applied instantaneously and distributed uniformly, so that each of the *N* pillars initially carries f=F/N. All pillars with strength thresholds σ<f fail immediately, releasing load that may trigger bursts of secondary failures. Depending on the adopted load-sharing rule and system parameters, these bursts may either terminate in a stable configuration or evolve into complete system collapse.

From a theoretical perspective, the redistribution of load released by a crushed pillar is determined by the adopted load-transfer protocol, which prescribes how the released load is distributed among the remaining intact pillars. In the literature, various load transfer schemes have been proposed to describe this process. Among them, two widely recognised mechanisms correspond to the limiting cases of load redistribution [14]. The first, known as global load sharing (GLS), assumes a perfectly rigid substrate, whereby the load released by a failed pillar is equally redistributed among all surviving elements in the system. Within this framework, all intact pillars are treated as equidistant in a mathematical sense, regardless of their actual spatial separation. Consequently, the GLS rule represents an idealised limit that is generally unrealistic for mechanical systems. At the opposite extreme lies the local load sharing (LLS) rule, which is physically more realistic as it assumes a substrate with finite compliance. Under this rule, the load from a failed pillar is redistributed only among its nearest intact neighbours, resulting in a short interaction range. Between these two limiting cases, several intermediate load transfer schemes have been proposed, including mixed-mode rules [15,16], power-law load transfer models [17,18,19], and fixed-range load sharing approaches [20,21]. These models interpolate between the global and local limits by introducing, respectively, a weighted combination of GLS and LLS contributions, a distance-dependent decay of transferred load, or a finite interaction radius that controls load localisation.

In pillar arrays, load redistribution is mediated by the elastic field induced in the substrate as a consequence of pillar crushing. Therefore, an appropriate load transfer rule should reflect this dynamic redistribution process and cannot be purely static, as assumed in the aforementioned models.

**Figure 1:**
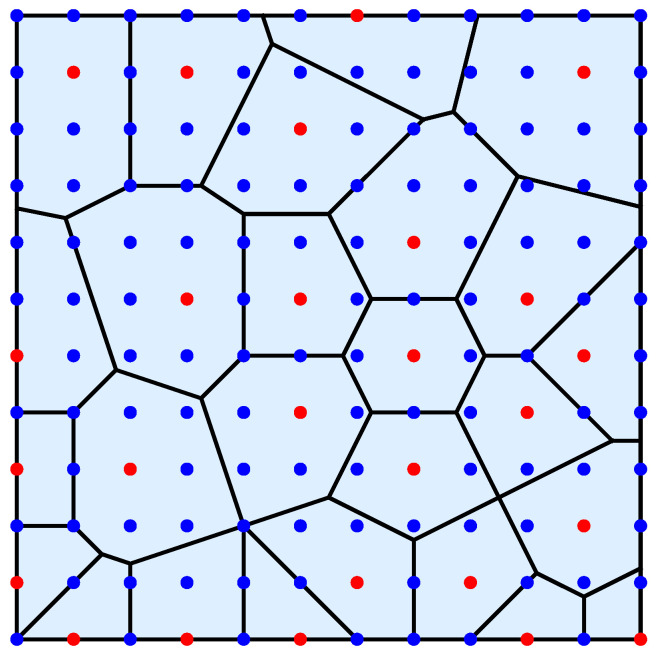
An exemplary Voronoi diagram for a system of 12×12 pillars: blue points represent intact pillars, red points represent pillars that failed simultaneously.

The present work introduces an alternative intermediate load transfer scheme based on the concept of Voronoi tessellation, which naturally accounts for spatial heterogeneity in load redistribution. This approach, referred to as Voronoi load sharing (VLS), defines the nodes hosting simultaneously failed pillars as the seeds of Voronoi cells (see Figure 1). The load carried by a failed pillar is then equally redistributed among all intact pillars within its corresponding Voronoi cell. Conceptually, the VLS rule represents an evolving mixture of the GLS and LLS schemes, as the effective load redistribution range changes dynamically with the spatial pattern of failures. In this way, the VLS rule effectively bridges the two limiting cases of global and local load sharing.

The primary objective of this study is to model and quantitatively characterise how the VLS rule governs system response under sudden loading conditions. Within our phenomenological framework, we restrict the analysis to a statistical characterisation of the system. Accordingly, the simulations are not based on nanoscale molecular dynamics [22,23] or submicron-scale continuum mechanics [24,25]. Instead, they operate on distributions of relevant microscopic quantities—such as pillar strength thresholds—to maintain a link between the externally applied load and the resulting local stresses experienced by individual pillars.

The paper is organised as follows: Section 2 introduces the model and computational methodology; Section 3 presents and discusses the simulation results; Section 4 outlines the limitations and perspectives; and Section 5 summarises the main findings.

## 2. Pillar Arrays Under Sudden Loading

The system under consideration consists of an array of *N* pillars, vertically arranged on a flat substrate. The geometrical configuration follows a square lattice, with N=L×L, where *L* denotes the linear size of the system. The array is subjected to axial compression, with the load magnitude rising abruptly from zero to a prescribed value.

Our minimal model incorporates two key components: (i) the load transfer rule, and (ii) a dichotomous functional relationship between the local load acting on a pillar and its individual strength threshold. In this formulation, each pillar is treated as a binary (intact–failed) element: it remains intact as long as the local load does not exceed its strength threshold, and fails irreversibly once this limit is surpassed. This binary rule enables a statistical description of the collective response while avoiding the explicit complexity of nanoscale mechanics.

In this section, we provide a detailed description of the modelling of the pillars and the sudden loading process.

### 2.1. Description of Arrays of Pillars

While the individual pillars are geometrically identical, they differ in their strength thresholds, reflecting the inherent fluctuations typical of micro- and nano-materials, as noted in the Introduction. These fluctuations are modelled by a random variable $\sigma_{th}$ which represents the strength threshold of each pillar, making the arrays a representative example of disordered systems [26,27].

In this work, we employ two types of probability distributions for $\sigma_{th}$: a uniform distribution defined on the interval [0,1], and a Weibull distribution, the latter being more physically grounded and widely used to model material failure [28,29,30].

For the Weibull distribution, the cumulative distribution function (CDF) in its two-parameter form reads

(1) $$P_{\eta ,\rho }\left(\sigma_{th}\right)=1-\exp\left[-{\left(\sigma_{th}/\eta \right)}^{\rho }\right]$$

where η and ρ denote the scale and shape parameters, respectively. In the present paper, we fix the scale parameter to η=1. The shape parameter ρ, commonly referred to as the Weibull index, governs the degree of disorder in the system. Specifically, increasing ρ reduces heterogeneity in the distribution of strength thresholds. In our model, the pillar strength thresholds are treated as quenched, meaning that neither temporal nor thermal fluctuations are taken into account.

### 2.2. Voronoi Tessellation for a Finite Set of Lattice Points and Its Interpretation in Dynamic Load Redistribution

Quite generally, consider a finite set of points $P=\left\{p_{1},p_{2},\cdots ,p_{k}\right\}$ selected from the sites of a lattice Λ embedded in the two-dimensional Euclidean space $R^{2}$. The Voronoi tessellation generated by P partitions the plane into a collection of convex polygonal regions ${\left\{V(p_{i})\right\}}_{i=1}^{k}$, where each region $V(p_{i})$ contains exactly one generating point $p_{i}$ and is defined as

$$V\left(p_{i}\right)=\left\{x\in R^{2}:∥x-p_{i}∥\leq ∥x-p_{j}∥,\forall j\neq i\right\},$$

with ∥·∥ denoting the Euclidean norm in $R^{2}$.

In this study, the conventional geometrical Voronoi construction is reformulated in dynamic terms by linking it to the propagation of elastic stress waves generated in the substrate as a result of pillar crushing. When a pillar fails, it emits a stress wave that travels radially outward through the substrate. Assuming isotropic elastic properties, the propagation velocity is uniform in all directions. Within this framework, the Voronoi cell $V(p_{i})$ associated with a crushed pillar at site $p_{i}$ represents the spatial region containing all intact pillars that are first reached by the elastic wavefront emitted from $p_{i}$, before wavefronts originating from any other simultaneously failing pillars. The cell boundaries therefore correspond to interfaces where stress waves from distinct failure sites meet, dynamically defining load-sharing zones across the substrate (see Figure 1). This interpretation connects the geometrical partitioning of space with the temporal sequence of stress-wave arrivals.

### 2.3. Voronoi Load Transfer Rule as an Evolving Mixture of GLS and LLS Rules

As stated above, in pillar arrays, the crushing of a pillar generates an elastic field within the underlying substrate. This field originates with the first local failure and continuously evolves as additional pillars collapse, reflecting the spatial distribution and clustering of damage. Consequently, a realistic load transfer rule should account for this dynamically evolving redistribution process.

To capture this behaviour, a Voronoi-diagram-based load-transfer rule is introduced. Under this scheme, the load previously supported by each failed pillar is redistributed equally among all intact pillars located within its corresponding Voronoi cell. In this way, the geometrical structure of the Voronoi tessellation naturally defines the spatial extent of load redistribution, while its dynamic evolution reflects the progressive nature of failure in the system.

As in the GLS rule, all intact pillars receive additional load when failures occur simultaneously. However, unlike GLS, the redistributed loads in VLS are not uniform. When failures are spatially dispersed, the resulting load field remains nearly homogeneous, and the system behaviour approaches that of the global sharing regime. In contrast, when failures occur in spatially clustered regions, the redistribution becomes strongly localised within these regions, and the effective load transfer range shortens, reproducing the characteristics of the LLS rule.

Hence, the VLS rule can be viewed as a dynamically evolving mixture of GLS and LLS mechanisms. Failure in VLS systems may therefore proceed through nucleation and cluster growth, similarly to the localised failure dynamics observed in LLS systems, rather than through the spatially uncorrelated, percolation-like failure typical of GLS systems. In fact, LLS systems are often considered closer to real materials, as failures tend to initiate and propagate through localised clusters of microcracks, reflecting the spatial correlations observed in experiments.

### 2.4. Subcritical and Critical Loads

The loading process can be implemented using one of three primary schemes: quasi-static, stepwise, or sudden. As we already briefly mentioned, in the quasi-static scheme, loading proceeds in an avalanche-like manner: between consecutive avalanches, the external load is increased just enough to trigger the failure of a single intact element. At the onset of each avalanche, the VLS rule becomes indistinguishable from the GLS rule. For the trivial case of a single seed, the Voronoi tessellation reduces to a single Voronoi cell encompassing the entire system. Since the GLS quasi-static loading process typically advances through long sequences of very small avalanches—followed only at the final stages by larger avalanches preceding global collapse—we largely omit the quasi-static scenario in our analysis. The second method, stepwise loading, requires choosing an appropriate step size [31]. If the step size is too small, the VLS rule again becomes indistinguishable from the GLS rule. On the other hand, a step size that is too large prevents an accurate determination of the system strength.

The appropriate loading scheme, enabling both a precise assessment of the system strength and a clear distinction between GLS and VLS behaviors, is the sudden loading scenario [32,33]. Compared to quasi-static loading, it more closely mimics the actual loading conditions typically encountered in practice. In the sudden loading scheme, the external load instantaneously raises from 0 to a specified value *F* and remains constant during the loading procedure. Nevertheless, for the sake of clarity, we emphasize that the loading protocol implemented in our numerical experiments is a theoretical construct rather than a representation of physical shock or impact loading. In order to compare results for different system sizes with respect to the strength thresholds of an individual pillar, we employ the quantity scaled by the system size, f=F/N. As the load *F* is applied uniformly on the initially intact and unloaded system, consequently the load per pillar is *f*.

Let $f_{c}$ denote the system’s critical load, i.e., the minimum load that triggers the complete collapse of the pillar array. When subjected to sudden loading, the array may attain one of the accessible stable configurations. A precritical load ($f_{\mathrm{pre}}<f_{c}$) can result in either no failures ($f_{\mathrm{pre}}<\min\left({\left\{\sigma_{th}^{i}\right\}}_{i=1}^{N}\right)$) or partial failure ($\min\left({\left\{\sigma_{th}^{i}\right\}}_{i=1}^{N}\right)\leq f_{\mathrm{pre}}<f_{c}$). In both cases, the integrity of the system is preserved and its load-bearing capacity is maintained. By contrast, a postcritical load ($f_{\mathrm{post}}\geq f_{c}$) leads to complete collapse of the array. Thus, a critically loaded array inevitably transitions from a state of local failure (microscopic damage) to a state of global failure (macroscopic damage), which is not observed under the subcritical regime. Our goal is to determine both $f_{c}$ and ultimate strength of the system $f_{\max}$, for each array. These quantities are related by $f_{c}=f_{\max}+\delta$, where δ>0 is vanishingly small. Here, $f_{\max}$ represents the maximum load the system sustains before complete collapse, i.e., its ultimate strength.

In order to approximate the values of $f_{c}$ and $f_{\max}$ we employ the bisection algorithm. The process is initialised with $f_{\mathrm{pre}}^{\left(0\right)}<f_{c}$ and $f_{\mathrm{post}}^{\left(0\right)}>f_{c}$. In the subsequent iterations, $f_{\mathrm{pre}}^{\left(n\right)}$ is increased and $f_{\mathrm{post}}^{\left(n\right)}$ is decreased according to the bisection algorithm, until the condition $f_{\mathrm{post}}^{\left(n\right)}-f_{\mathrm{pre}}^{\left(n\right)}<\delta$ is satisfied, where $\delta =\min\left(10^{-4},N^{-1}\right)$. Finally, we obtain the approximations $f_{c}\approx \min\left(\left\{f_{\mathrm{post}}^{\left(n\right)}\right\}\right)$ and $f_{\max}\approx \max\left(\left\{f_{\mathrm{pre}}^{\left(n\right)}\right\}\right)$.

### 2.5. Relaxation Time

Under sudden loading, the failure process evolves through successive bursts of pillars that fail simultaneously and instantaneously. In numerical terms, simultaneity is defined at the iteration level: all pillars whose local loads exceed their individual strength thresholds within the same redistribution step are assumed to fail concurrently. Initially, at time t=0, we consider an undamaged array with *N* intact pillars. The sequence of the numbers of pillars crushed at *j*-th time (iteration) step can be denoted as $d_{t=1},d_{t=2},\ldots ,d_{t=\tau }$, where τ represents the relaxation time of the system, i.e., the total number of bursts after which the system reaches a stable configuration. The value of τ, like $f_{c}$ and $f_{\max}$, is a random variable. In our sudden loading procedure, each load redistribution corresponds to one time step, so that the total number of load redistribution steps equals τ−1 in the postcritical state and τ in the precritical state. If the system undergoes complete collapse under loading, then τ coincides with the system’s failure time, $\tau_{f}$. The cascade-like failure process governed by the VLS rule is inherently dynamic. As the number of failed pillars changes from one iteration to the next, both the number of Voronoi cells and the positions of their generating seeds evolve. This leads to continuous modifications of the Voronoi tessellation, reflecting the evolving load-sharing topology. The impact of this dynamics on the overall behaviour of the system will be discussed in the next section. Results for the GLS and LLS rules are provided for comparison.

## 3. Results of Simulations of Suddenly Loaded Arrays Under the VLS, in Comparison with Results Obtained for the GLS and LLS

Based on the model described in Section 2, we have developed a computational framework designed to simulate the failure processes of pillar arrays under the application of the GLS, LLS, and VLS rules.

We focus on systems subjected to sudden loading, with load values representing both subcritical and critical regimes. This approach makes it possible to systematically analyse the temporal evolution of the number of crushed pillars, providing detailed insight into the dynamics of successive bursts of failures in both regimes. In particular, it allows us to capture differences in failure progression and load redistribution mechanisms that emerge under distinct load transfer rules.

System sizes *N* were chosen to be sufficiently large to capture possible size-dependent effects. The role of material defects was investigated by considering different distributions of pillar strength thresholds $\sigma_{th}$, specifically a uniform distribution and Weibull distributions with a few selected shape parameters ρ=2,5,8 corresponding to increasing levels of homogeneity in $\sigma_{th}$.

To ensure reliable statistics, the failure process for each system size *N* and each distribution of $\sigma_{th}$ was simulated over $M=10^{4}$ statistically independent realisations of pillar arrays.

### 3.1. Strength of the System

In analysing the simulation results, we begin with the system strength $f_{\max}$. It is well established that under the GLS rule, the mean system strength asymptotically converges to a finite, non-zero value [10,34,35]. For large systems (N≫1), with strength thresholds $\sigma_{th}$ uniformly distributed on the interval $\left[0,1\right]$, the mean strength is given by

(2) $$\langle f_{\max}^{\mathrm{GLS}}\left(N,\mathrm{uniform}\right)\rangle =\frac{1}{4}\left(1+1.24528\;N^{-2/3}\right).$$

In the case where the strength thresholds follow a Weibull distribution, the corresponding quantity reads [36]

(3) $$\langle f_{\max}^{\mathrm{GLS}}\left(N,\rho \right)\rangle ={\left(\rho \;e\right)}^{-1/\rho }\left[1+0.996\cdot {\left(\frac{e^{2/\rho }}{\rho }\right)}^{1/3}\cdot N^{-2/3}\right].$$

The GLS rule represents a mean-field approximation. Within the global load-sharing framework, the system strength, together with the associated critical load, is independent of the specific loading protocol.

In contrast, when the LLS rule is employed, the results differ significantly. Systems subjected to sudden loading under the LLS rule exhibit lower values of $f_{\max}$ and $f_{c}$ compared to those obtained under quasi-static loading conditions. Generally, it is known that $f_{c}∼1/\ln N$ [11,28]. The mean strength of LLS systems under sudden loading is well approximated by the following size-dependent formula [19,37]

(4) $$\langle f_{\max}^{\mathrm{LLS}}\left(N\right)\rangle =\frac{\alpha }{{\left(\ln N\right)}^{\beta }}$$

where α and β are fitting parameters. The values of these parameters, obtained from our simulations for different system disorders, are summarised in Table 1. A pronounced size effect is evident in LLS systems: the mean strength, $\langle f_{\max}^{\mathrm{LLS}}\left(N\right)\rangle$, decreases monotonically toward zero as *N* keep growing. Table 1 also reports the root mean square error (RMSE) as an indicator of fit quality. The consistently low RMSE values across all distributions of pillar strength thresholds confirm the accuracy of the fits. These supplementary statistics for the LLS systems are included both to validate the reliability of the estimated values and to provide a reference point for comparison with the results of our Voronoi-based model.

It turns out that the size-effect in the VLS rule is correctly approximated by the following formula:

(5) $$\langle f_{\max}^{\mathrm{VLS}}\left(N\right)\rangle =\frac{\alpha }{{\left(\ln N\right)}^{\beta }}\cdot \left(1+\gamma /N\right).$$

This formula extends Equation (4), which properly describes LLS systems, by introducing the factor, (1+γ/N), thus incorporating an additional parameter. The fitted values of the parameters α, β and γ, together with the measure of the fit (RMSE), are presented in Table 1. The values of the coefficients α and β are not the same for the VLS and LLS rules. This indicates that results generated by the VLS rule cannot be obtained by multiplying those obtained under the LLS rule by a simple multiplication by the factor (1+γ/N).

**Figure 2:**
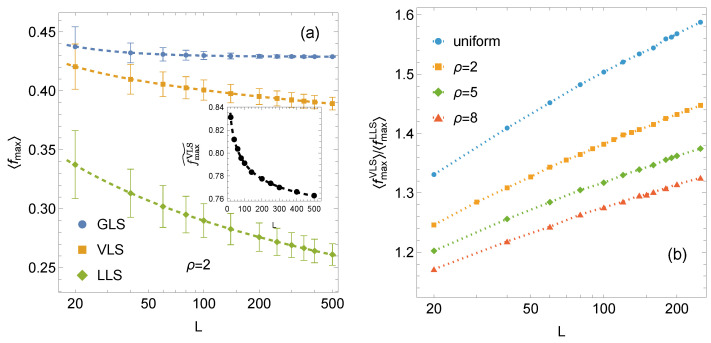
(**a**) Log-linear plot of the mean strength for arrays of L×L pillars, comparing GLS, VLS, and LLS systems. The GLS, LLS, and VLS curves are fitted using Equations (3), (4), and (5), respectively. Error bars indicate ±1 standard deviation computed from $10^{4}$ samples. Inset: normalised relative strength of VLS systems, as defined in Equation (6). (**b**) Log-linear plot of the ratio $\langle f_{\max}^{\mathrm{VLS}}\rangle /\langle f_{\max}^{\mathrm{LLS}}\rangle$) for arrays of L×L pillars for the considered distributions of $\left\{\sigma_{th}\right\}$.

A graphical comparison of the mean system strength under the GLS, VLS, and LLS rules is shown in Figure 2. The VLS curve lies between the two limiting cases. The $\langle f_{\max}^{\mathrm{VLS}}\left(N\right)\rangle$ curve decreases monotonically toward zero, similarly to the LLS case, but at a considerably slower rate. Within the examined range of system sizes, the VLS results remain closer to the GLS curve, yet their asymptotic trend clearly differs from that of GLS, which does not decay to zero. Therefore, it is instructive to examine the relative strength of the VLS systems which we define as

(6) $$\tilde{f_{\max}^{\mathrm{VLS}}}\left(N\right)=\frac{\langle f_{\max}^{\mathrm{VLS}}\left(N\right)\rangle -\langle f_{\max}^{\mathrm{LLS}}\left(N\right)\rangle }{\langle f_{\max}^{\mathrm{GLS}}\left(N\right)\rangle -\langle f_{\max}^{\mathrm{LLS}}\left(N\right)\rangle }.$$

As shown in the inset in Figure 2, the relative strength $\tilde{f_{\max}^{\mathrm{VLS}}}$ decreases with increasing system size signalising that VLS systems gradually approach the behavior of LLS systems as *N* grows. At the same time, the ratio $\langle f_{\max}^{\mathrm{VLS}}\left(N\right)\rangle /\langle f_{\max}^{\mathrm{LLS}}\left(N\right)\rangle$ increases with system size (see Figure 2b). These trends together indicate that, although VLS systems exhibit a size effect, it is significantly weaker than that observed for LLS systems.

**Figure 3:**
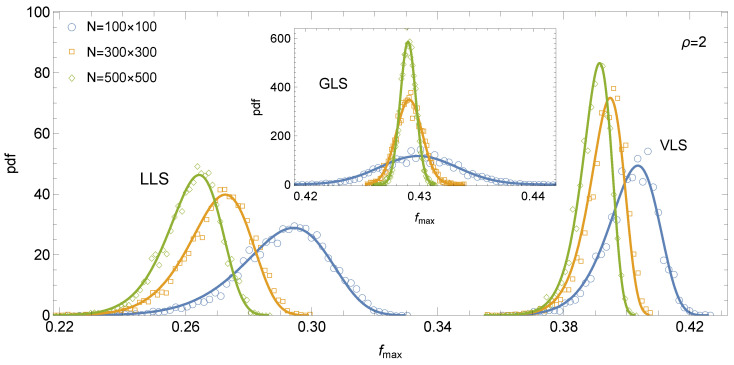
Empirical distributions of $f_{\max}$ for increasing system sizes *N* and different load-sharing rules. Pillar strength thresholds follow Equation (1), with ρ=2. Solid lines represent Equation (7), with parameters estimated from the data. The inset shows the corresponding distributions for the GLS rule, where the solid line follows the normal probability density function.

Analysis of the empirical distributions of system strength reveals clear distinctions among the three load-sharing rules. For GLS systems, the distribution of $f_{\max}^{\mathrm{GLS}}$ is symmetric and well approximated by a Gaussian law (see inset in Figure 3). In contrast, LLS systems display a moderately left-skewed distribution, which is accurately described by the three-parameter Weibull distribution (see Figure 3). The VLS rule exhibits a qualitatively similar trend to LLS, as the distribution of $f_{\max}^{\mathrm{VLS}}$ is also negatively skewed. Interestingly, the skewness in VLS systems, particularly for larger *N*, tends to exceed that observed in LLS systems, although it generally remains within the range of moderate skewness. Consequently, the distribution of $f_{\max}^{\mathrm{VLS}}$—similarly to that of $f_{\max}^{\mathrm{LLS}}$—is modelled using the three-parameter Weibull distribution (see Figure 3)

(7) $$p_{k,\xi ,\theta }\left(f_{\max}\right)=\frac{k}{\xi }{\left(\frac{f_{\max}-\theta }{\xi }\right)}^{k-1}\exp\left[-{\left(\frac{f_{\max}-\theta }{\xi }\right)}^{k}\right],\;f_{\max}\geq \theta ,$$

where k,ξ,θ are the shape, scale, and location parameters, respectively.

It is worth emphasising that selecting an appropriate distribution for the collected data is a crucial step. Several established methods are available for constructing confidence intervals and performing hypothesis tests, including the universal framework [38]. With this in mind, the data sets were thoroughly examined using appropriate goodness-of-fit tests, through which we identified $p_{k,\xi ,\theta }\left(\cdot \right)$, seen in Equation (7), as the distribution that most accurately represents the empirical distributions of $f_{\max}^{\mathrm{VLS}}$. It should be noted, however, that a substantial portion of the data can also be satisfactorily described by the three-parameter skew-normal probability distribution. Nevertheless, we chose to represent all data using Equation (7) because the Weibull distribution provides an adequate fit for nearly all data sets and, in cases where both models are acceptable, yields higher maximised likelihood values and greater *p*-values than the skew-normal model.

A comparison of the corresponding probability density functions highlights a pronounced size effect in VLS systems: with increasing system size, the pdf shifts leftwards while its peak grows, reflecting decreasing mean strength and reduced dispersion. This trend closely resembles the behaviour observed in LLS systems. In contrast, GLS systems display virtually no size effect in terms of mean strength, exhibiting only a reduction in dispersion with increasing system size.

### 3.2. Fraction of Pillars Failing Under Subcritical Load $f_{\max}$

When a subcritical load $f_{\max}$ is applied to an array of *N* pillars, a certain fraction of pillars fails. Let U(N) denote the fraction of intact pillars that remain capable of sustaining the load $f_{\max}$. The complementary fraction $\bar{U}\left(N\right)=1-U\left(N\right)$ therefore represents the proportion of crushed pillars. An important question is how $\bar{U}$ scales with system size *N*, that is, how large the fraction of failed pillars can become while the system remains marginally stable under the applied load.

For GLS systems, there exists an analytical solution to this question. Specifically, the fraction of failed pillars under $f_{\max}$ asymptotically converges to a nonzero value in the limit N→∞:

(8) $$\bar{U}\underset{N\to \infty }{\to }1-e^{-1/\rho }$$

for systems with Weibull-distributed strength thresholds, whereas for systems with uniformly distributed thresholds $\sigma_{th}$ the corresponding asymptotic value is

(9) $$\bar{U}\underset{N\to \infty }{\to }\frac{1}{2}.$$

The quantity $\bar{U}$ can be interpreted as a subcritical fraction, as it represents the largest proportion of pillars that may fail without triggering global failure when the applied load remains below the critical load $f_{c}$. In this sense, $\bar{U}$ characterises the maximum extent of local failure that the array can tolerate under sudden loading while still preserving overall stability.

Based on the simulation results, we find that the mean subcritical fraction $\langle \bar{U}\left(N\right)\rangle$ for both LLS and VLS systems is accurately captured by the empirical relation

(10) $$\langle \bar{U}\left(N\right)\rangle =\frac{\omega }{{\left(\ln N\right)}^{\lambda }}\cdot \left(1+\frac{\kappa }{\sqrt{N}}\right)$$

where the parameters ω, λ, and κ are determined by fitting the expression to the numerical data. The corresponding parameter estimates for LLS and VLS systems are summarised in Table 2.

**Figure 4:**
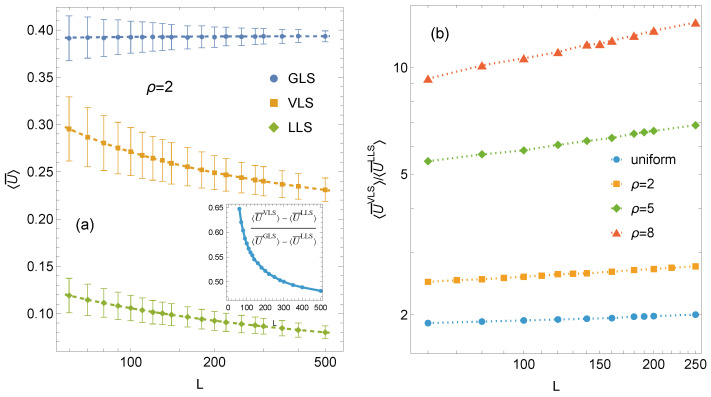
(**a**) Mean subcritical fraction of failed pillars, $\langle \bar{U}\rangle$, for arrays with L×L pillars. Error bars indicate ±1 standard deviation computed from $10^{4}$ samples. Inset: the relative VLS mean fraction of failed pillars. (**b**) The variation of ratio $\langle {\bar{U}}^{\mathrm{VLS}}\rangle /\langle {\bar{U}}^{\mathrm{LLS}}\rangle$ for different distributions of pillar strength thresholds.

The dependence of the mean subcritical fraction of failed pillars on system size for GLS, VLS, and LLS systems is presented in Figure 4a. Analysis of the fitted curves reveals that the VLS rule represents an intermediate load-sharing scheme, although its trend closely follows that of the LLS system. Both curves decrease monotonically with increasing system size *N*. In contrast, GLS systems display virtually no size effect—the mean subcritical fraction increases only slightly, reflecting the diminishing statistical fluctuations that accompany larger *N*. In absolute terms, the mean subcritical fraction in VLS systems decreases more rapidly than in LLS systems, and the behaviour of VLS systems gradually converges toward that of LLS systems as *N* increases (see inset in Figure 4a). However, when relative values are considered, the LLS curve decays faster than the VLS curve, as shown in Figure 4b, which presents the ratio $\langle {\bar{U}}^{\mathrm{VLS}}\rangle /\langle {\bar{U}}^{\mathrm{LLS}}\rangle$. This ratio increases with system size and becomes larger for weaker disorder. The latter observation reflects the tendency of LLS systems to exhibit increasingly brittle behaviour as disorder decreases. In the ideal brittle limit, any local failure triggers a self-sustaining cascade of bursts leading to complete system collapse, and consequently $\bar{U}=0$ under $f_{\max}$.

The corresponding mean subcritical fractions of failed pillars are summarised in Table 3. Owing to its perfectly homogeneous load redistribution, the GLS rule allows the largest fraction of failed pillars, resulting in the smallest proportion of surviving ones and the highest maximum load $f_{\max}$ among the three schemes. Conversely, LLS systems sustain the lowest $f_{\max}$ and exhibit the smallest subcritical fraction. The VLS rule, as expected, lies between those two limiting cases. Although the load redistribution in VLS systems is not uniform, the subcritical fraction of failed pillars remains significantly higher than in LLS systems across all analyzed sizes. This contrast becomes particularly pronounced in weakly disordered systems (ρ=5 and ρ=8), where LLS systems become close to the brittle limit ($\bar{U}≳0$), whereas VLS systems maintain values several times higher. For ρ=8, in particular, the LLS response is nearly perfectly brittle, allowing only isolated failures under $f_{\max}$, while the corresponding VLS values exceed them by more than an order of magnitude (see Figure 4b).

### 3.3. Relaxation Time and Evolution of the Number of Failed Pillars

The loading process evolves through a sequence of failure bursts that occur between the initially intact configuration and the final stable configuration attained after loading. The total number of these bursts defines the relaxation time τ, as introduced in Section 2.5. Consequently, the sequence of bursts provides the distribution of failure sizes, representing the number of pillars that fail at each successive step.

**Figure 5:**
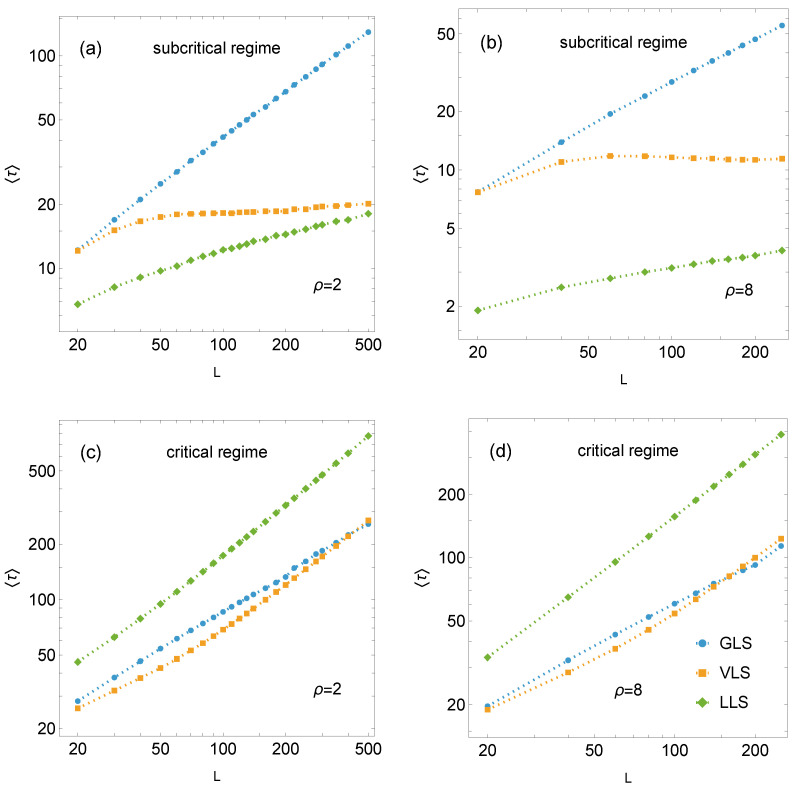
Log-log plots of the mean relaxation time for pillar arrays of size L×L under GLS, VLS, and LLS rules. Panels (**a**,**b**) correspond to the subcritical loading, while panels (**c**,**d**) represent the critical loading regime. Simulation results are shown as data points; dashed lines are included as visual guides.

Figure 5 presents the mean relaxation times as a function of the linear system size *L* for systems subjected to the subcritical load $f_{\max}$ (panels (a) and (b)) and the critical load $f_{c}$ (panels (c) and (d)).

In the subcritical regime, the relaxation time of GLS systems is longer than that for the VLS and LLS systems, reflecting a greater number of bursts before reaching a stable configuration of partial failure. Within the analysed range of system sizes, the damage process is shortest in LLS systems, while the relaxation time for VLS systems lies between the two extremes. The mean relaxation time increases with system size for both GLS and LLS systems, although this growth is noticeably slower for the latter. The VLS rule exhibits a distinct pattern: for ρ=2 and uniform disorder, the mean relaxation time rises more slowly than in the local case, whereas for ρ=5 and ρ=8, it becomes almost insensitive to system size. Consequently, for small to moderately large system sizes, the mean relaxation time of VLS systems resembles that of GLS systems and gradually approaches that of LLS systems as the system size increases.

A different pattern emerges in the critical regime (Figure 5c,d). Here, LLS systems display markedly longer mean relaxation times than those observed for VLS and GLS systems. Interestingly, for most system sizes (except the largest), the critical failure process in VLS systems terminates in fewer bursts than in the GLS case. However, beyond a certain size – depending on the strength-threshold distribution – $\langle \tau_{f}^{\mathrm{VLS}}\left(L\right)\rangle$ grows faster than $\langle \tau_{f}^{\mathrm{GLS}}\left(L\right)\rangle$, and for the largest simulated systems, the GLS rule yields the shortest relaxation time.

**Figure 6:**
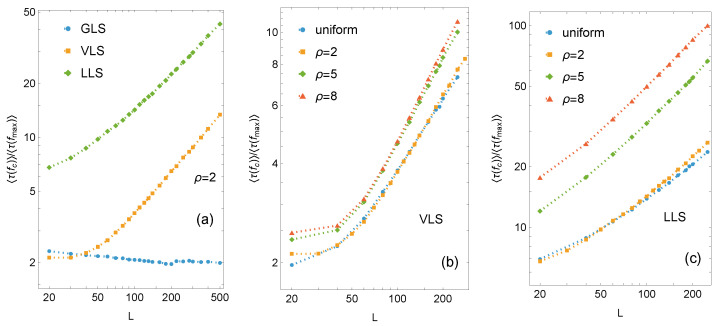
Variation of the ratio $\langle \tau (f_{c})\rangle /\langle \tau (f_{\max})\rangle$ for arrays with L×L pillars: (**a**) comparison among GLS, VLS, and LLS systems, (**b**) comparison for different pillar strength threshold distributions for VLS systems, and (**c**) analogous comparison for LLS systems.

The ratio $\langle \tau (f_{c})\rangle /\langle \tau (f_{\max})\rangle$ is shown in Figure 6. For GLS systems, this ratio remains approximately 2, with only minor statistical fluctuations across all strength distributions. Thus, the mean relaxation time under subcritical loading is roughly half that under the critical load. In contrast, a pronounced size effect is observed for LLS systems, where the ratio $\langle \tau^{\mathrm{LLS}}\left(f_{c}\right)\rangle /\langle \tau^{\mathrm{LLS}}\left(f_{\max}\right)\rangle$ increases rapidly with system size and clearly depends on the type of disorder (Figure 6c). Voronoi-based systems also show dependence on system size and disorder, though both effects are far less pronounced than in LLS systems (Figure 6b). Hence, the VLS behavior is clearly distinct from the global scheme but tends toward LLS dynamics while remaining less localised.

A closer look at LLS systems reveals a sharp contrast between their subcritical ($f_{\max}$) and critical ($f_{c}$) responses. Under $f_{\max}$, they rapidly reach a stable configuration (short relaxation time) with a low subcritical fraction $\bar{U}$ of failed pillars (see Table 3). However, under the critical load $f_{c}$, which exceeds $f_{\max}$ only by a small increment δ, all pillars fail in a prolonged, self-sustaining catastrophic avalanche of bursts. Thus, the critical load $f_{c}$ represents a bifurcation point at which the system transitions from a stable, partially failed configuration with residual load-bearing capacity to one of complete global failure.

Even though VLS systems exhibit size effects similar to those observed in LLS systems, the transition at the bifurcation point is less pronounced. This behaviour results from two factors: (i) the subcritical fraction $\bar{U}$ is considerably larger than in LLS systems, and (ii) the associated catastrophic avalanche is noticeably shorter, as it develops over a substantially smaller number of bursts.

**Figure 7:**
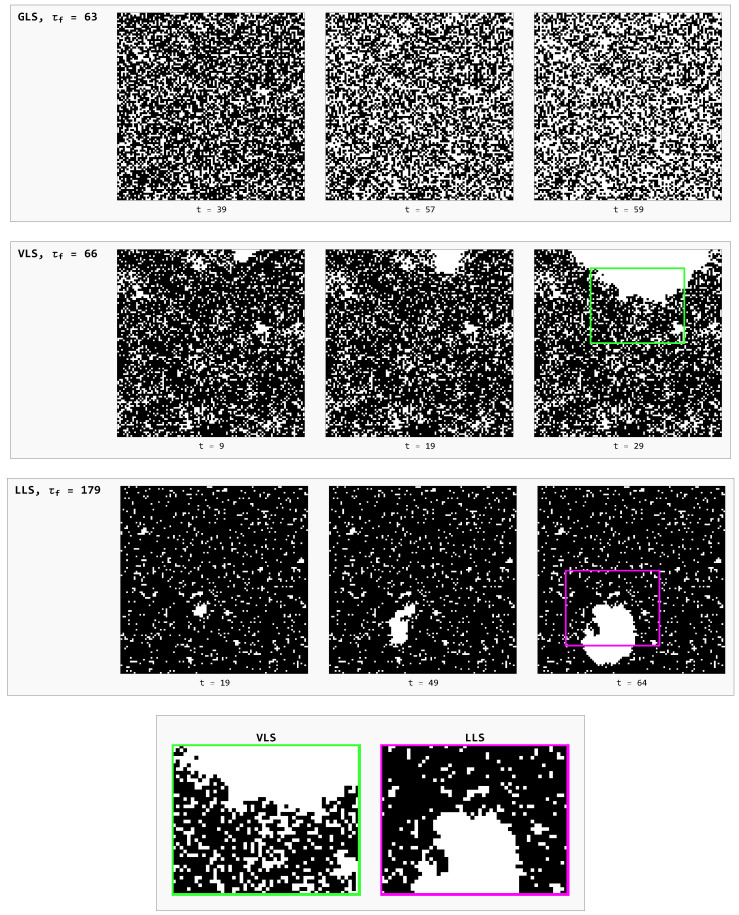
Snapshots of the failure process for arrays of 100×100 pillars at selected time steps, comparing GLS, VLS, and LLS systems. Pillar strength thresholds $\left\{\sigma_{th}\right\}$ follow the Weibull distribution given by Equation (1), with ρ=2. Intact pillars are shown as black squares, while failed pillars are represented by white (missing) squares. The bottom row presents magnified views of selected regions for the VLS and LLS cases, highlighting the local morphology of failure clusters.

It is instructive to directly examine the spatio-temporal evolution of the failure process under critical loading for all considered load-sharing rules. This approach allows us to visualise how pillar crushing propagates through the array, as the system evolves from a state of localised, individual pillar failures (microscopic failure) to global system collapse (macroscopic failure). Figure 7 shows snapshots of representative GLS, VLS, and LLS systems at selected time steps under $f_{c}$.

In GLS systems, the failure process is entirely random throughout its evolution—failures of pillars occur at random locations across the array (top row in Figure 7). In this case, both the spatial arrangement of the pillars and the system geometry are irrelevant, as the GLS rule corresponds to a perfectly rigid substrate. The density of failed elements after the first time step (not included in Figure 7) is the highest among all models, since the largest critical load $f_{c}$ is required to initiate the catastrophic avalanche.

Concerning the snapshot of the LLS system (third row in Figure 7), the onset of global failure occurs at the lowest critical load $f_{c}$, the fraction of failed elements at the initial step is minimal, and the degree of initial damage remains low. The first failures are randomly scattered throughout the system, as only pillars with $\sigma_{th}<f_{c}$ collapse. Subsequently, due to fully localised load redistribution, further failures predominantly occur in the neighbourhoods of previously crushed pillars, forming numerous small clusters. This process quickly stabilizes, leading to the nucleation of a single dominant cluster (see the white region slightly below and to the left of the centre at t=49 in the third row of Figure 7), which then expands over successive time steps by incorporating smaller clusters and ultimately drives global failure. Owing to the strong spatial correlations between local failures, the destruction propagates as a coherent wave with a smooth front, reflecting the initially low density of failed pillars prior to the growth of the dominant cluster.

Although the spatial evolution of failures in VLS model, as seen in the second row in Figure 7, partially resembles that of LLS systems, there are significant quantitative and qualitative differences. In particular, the catastrophic avalanche in the VLS model is triggered at a substantially higher critical load than in the LLS case. Consequently, a considerably larger fraction of pillars becomes damaged already after the initial time step, initiating a burst of secondary failures in subsequent steps. These secondary failures tend to coalesce into significantly larger clusters than those observed under the LLS rule. Similar to the LLS case, a dominant cluster eventually emerges in VLS systems; however, its formation occurs at a later stage of avalanche development. Furthermore, because load transfer in the VLS rule is less localised, failures are not confined solely to the dominant cluster. The resulting destruction front in VLS systems is therefore rough and irregular, in sharp contrast to the smooth, well-defined front characteristic of LLS systems (see bottom row of Figure 7).

From a temporal perspective, the evolution of critically loaded VLS systems, measured in terms of relaxation time, closely resembles that of GLS systems. Taken together, the spatio-temporal characteristics reveal that the VLS rule combines features of both LLS and GLS models. In particular, critically loaded VLS systems exhibit an interplay between the critical load—substantially higher than in LLS yet lower than in GLS—and the effective range of load redistribution, which is neither fully localised nor entirely global. This interplay produces relaxation times that are significantly shorter than in LLS systems and approach those of GLS systems, despite the much higher critical load observed in the latter. The underlying reason is that failure progression in VLS systems remains cluster-like, in contrast to the cluster-free dynamics of GLS systems, where redistributed load is shared equally among all surviving pillars.

**Figure 8:**
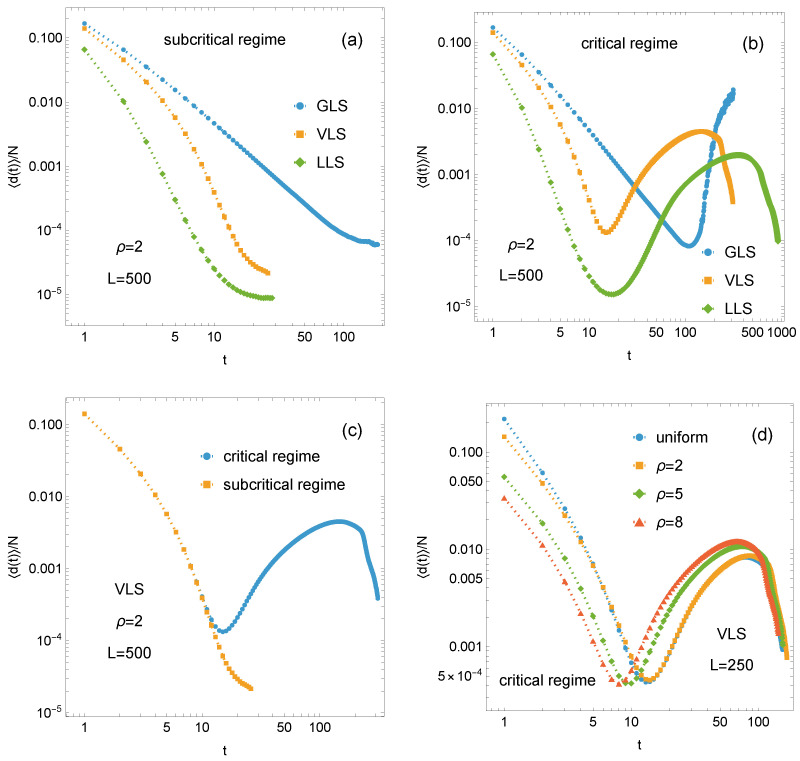
Evolution of the mean fraction of failed pillars: (**a**) subcritical loading $f_{\max}$—comparison of GLS, VLS, and LLS rules, (**b**) critical loading $f_{c}$—comparison of GLS, VLS, and LLS rules, (**c**) comparison of VLS systems under critical and subcritical loading, (**d**) VLS systems under critical loading for different distributions of $\left\{\sigma_{th}\right\}$.

Finally, we examine the evolution of the failure process, averaged over all samples for each configuration. Figure 8 presents the mean fraction of failed pillars at successive time steps as a function of time for both the subcritical and critical regimes. The following observations are worth noting:

(i) The results for the subcritical regime are shown in Figure 8a. It is observed that the curves of 〈d(t)〉/N decrease with time *t* for all load-sharing rules, with the most rapid decline occurring in the LLS systems. The mean number of failed pillars decreases monotonically, particularly in the LLS and VLS systems, until the failure process ceases. The intermediate nature of the VLS rule is clearly visible, as the corresponding 〈d(t)〉/N curve consistently lies between the two limiting cases represented by the GLS and LLS systems.

(ii) Figure 8b presents the mean fraction of failed pillars at successive time steps under critical loading. Initially, 〈d(t)〉/N decreases for all load-sharing rules. However, unlike in the subcritical regime, the curves exhibit an upturn once a minimum is reached. For GLS systems, this upturn develops into a monotonic increase that continues until global failure occurs. In contrast, VLS and LLS systems display a distinct pattern: after reaching their global minima, 〈d(t)〉/N increases toward a local maximum and subsequently decreases during the final stage of failure. Despite quantitative differences, such as in relaxation times, the LLS and VLS curves exhibit qualitatively similar behaviour. Panel (c) of Figure 8 combines the information presented in panels (a) and (b), focusing exclusively on the VLS systems.

(iii) Regardless of whether the system is in the subcritical or critical regime, the early-stage dynamics of VLS and, in particular, LLS systems are characterised by a rapid decrease in the fraction of failed pillars, with no apparent distinction between the two regimes, as shown in panels (a), (b), and (c) of Figure 8. Divergence appears only near the minimum of the critical-regime curve: while the subcritical curve continues to fall steeply, the critical curve slows, signalling imminent system collapse. Importantly, the characteristic shape of the critical-regime VLS curve in Figure 8b is independent of the assumed strength-threshold distribution. The same qualitative behaviour is observed for both the uniform case and for Weibull distributions with ρ=2, 5, and 8 (see Figure 8d).

## 4. Limitations and Perspectives

The results presented in this work are derived from a statistically grounded modelling framework that requires extensive numerical simulations. This reliance on computational approaches is justified by the intrinsic complexity of the problem: analytically tractable solutions are not available beyond mean-field approximations such as the GLS fibre-bundle model.

A further limitation stems from the phenomenological nature of the proposed VLS rule. While it incorporates key geometric and elastic features, a fully resolved description of substrate mechanics would require a more detailed continuum-mechanics treatment, which lies beyond the scope of the present study.

Additionally, our analysis focuses exclusively on sudden loading. Real engineering systems may experience a broader spectrum of loading modes, including, for example, cyclic or rate-dependent loading, which could influence both load redistribution and damage accumulation and should therefore be investigated in future work.

Finally, modern micropillar arrays are integral components of a wide range of nanoscale devices, such as piezoelectric nanogenerators and Cu-pillar bump arrays used in flip-chip packaging technology. Although numerous studies on the compression of individual mesoscale pillars have been reported, there is, to the best of our knowledge, a conspicuous lack of publications addressing compression experiments on micropillar arrays, even for configurations comprising only a modest number of units. Consequently, a direct comparison between our findings and existing experimental observations is currently not feasible.

It remains an open question how the load-transfer framework developed here can be adapted to describe failure in systems where substrate heterogeneity may strongly influence local load redistribution. Nevertheless, pillar-bump arrays may offer a promising platform for experimental investigations of cascading failure phenomena. Such arrays, which frequently consist of hundreds of thousands of micrometre-scale pillars on a single die, can undergo substantial stress accumulation and fatigue, particularly during thermal cycling. These effects may lead to multiple failure modes, including cascade-like fracture events.

Addressing these issues represents a direction for future research and would enhance the applicability of Voronoi-based load-sharing models in the reliability analysis of advanced micro- and nanoscale mechanical systems.

## 5. Summary

We have numerically investigated the damage evolution in suddenly loaded pillar arrays, where the load redistribution follows a Voronoi-diagram-based scheme generated by simultaneously failed pillars. The proposed Voronoi load sharing (VLS) rule was examined in comparison with the two limiting load-sharing mechanisms: global load sharing (GLS) and local load sharing (LLS).

Simulations were performed in two regimes: (i) a subcritical regime, in which the failure process stabilises after a finite sequence of bursts, leaving the system partially damaged, and (ii) a critical regime, characterised by a self-sustained catastrophic avalanche of pillar failures leading to complete array collapse. These analyses enabled the evaluation of several global system characteristics—mean strength, critical load, subcritical fraction of failed pillars, and relaxation time—together with a detailed examination of the spatio-temporal evolution of failure. The results demonstrate that the VLS rule constitutes an intermediate load-transfer mechanism that effectively bridges the idealised GLS and LLS schemes. The obtained values of the system strength, critical-regime relaxation time, and subcritical fraction of failed pillars more closely resemble those computed for GLS systems than those observed in LLS systems. However, the pronounced size effects, asymmetric shape of the system strength distribution, and scaling of subcritical damage are more characteristic of LLS systems.

The failure dynamics in VLS systems are nucleation-driven, with localised clusters of failed pillars forming and growing in space, similar to the LLS behaviour. This contrasts with the percolation-like, spatially uncorrelated failure typical of GLS systems. Moreover, the temporal evolution of failure events in VLS follows a progression that closely resembles that of LLS systems.

In conclusion, the Voronoi-diagram-based load-transfer rule provides a physically grounded and dynamically evolving framework for modelling load redistribution in parallel multicomponent mechanical systems. It captures key features of both global and local load-sharing regimes, offering a realistic intermediate description of failure progression and collective response in pillar arrays. Our phenomenological approach may serve as a basis for future experimental investigations of micro- and nano-pillar arrays.

## Figures and Tables

**Table 1:** Estimated values of fitted parameters for the mean system strength in VLS arrays (Equation (5)) and LLS arrays (Equation (4)), along with RMSE.

Distribution of $\sigma_{th}$	Model	Parameters			RMSE
		α	β	γ	
uniform	VLS	0.293	0.122	4.430	0.000059
	LLS	0.383	0.426	—	0.000131
ρ=2	VLS	0.497	0.097	2.946	0.000111
	LLS	0.631	0.351	—	0.000217
ρ=5	VLS	0.661	0.066	0.530	0.000112
	LLS	0.819	0.287	—	0.000367
ρ=8	VLS	0.746	0.054	−0.017	0.000072
	LLS	0.917	0.257	—	0.000600

**Table 2:** Estimated values of the fitted parameters ω, λ, κ for the mean subcritical fraction of failed pillars, $\bar{U}$, in VLS and LLS systems (Equation (10)), along with RMSE.

Distribution of $\sigma_{\mathrm{th}}$	Model	Parameters			RMSE
		ω	λ	κ	
uniform	VLS	0.5610	0.2204	5.0393	0.000261
	LLS	0.6174	0.5434	1.4444	0.000162
ρ=2	VLS	0.5356	0.3402	7.6723	0.000146
	LLS	0.6533	0.8354	3.4170	0.000164
ρ=5	VLS	0.3214	0.5450	11.2535	0.000133
	LLS	1.0974	1.8383	1.7728	0.000035
ρ=8	VLS	0.3533	0.7993	10.2371	0.000104
	LLS	1.0471	2.3169	1.7515	0.000030

**Table 3:** Mean subcritical fraction of failed pillars, $\bar{U}$, for GLS, VLS, and LLS systems, evaluated for selected system sizes N=L×L, and shown with respect to the employed distributions of pillar strength thresholds.

$\bar{U}$				
Load Sharing Rule	L=60	L=100	L=200	L=250
**uniform**				
GLS	0.4979	0.4983	0.4986	0.4996
VLS	0.3823	0.3616	0.3418	0.3370
LLS	0.2016	0.1874	0.1723	0.1682
ρ=2				
GLS	0.3912	0.3923	0.3924	0.3928
VLS	0.2953	0.2713	0.2490	0.2438
LLS	0.1191	0.1059	0.0924	0.0889
ρ=5				
GLS	0.1792	0.1801	0.1805	0.1807
VLS	0.1214	0.1063	0.0937	0.0907
LLS	0.0223	0.0182	0.0141	0.0132
ρ=8				
GLS	0.1161	0.1165	0.1168	0.1171
VLS	0.0769	0.0662	0.0563	0.0541
LLS	0.0083	0.0062	0.0044	0.0040

## Data Availability

The original contributions presented in this study are included in the article. Further inquiries can be directed to the corresponding author.
